# Case Report: Vemurafenib Treatment in Brain Metastases of *BRAF^S365L^*-Mutant Lung Papillary Cancer by Genetic Sequencing of Cerebrospinal Fluid Circulating Tumor DNA Detection

**DOI:** 10.3389/fonc.2021.688200

**Published:** 2021-06-11

**Authors:** Jianing Jiang, Jinqi Gao, Gang Wang, Jinyan Lv, Wenting Chen, Jing Ben, Ruoyu Wang

**Affiliations:** ^1^ Department of Oncology Medicine, Affiliated Zhongshan Hospital of Dalian University, Dalian, China; ^2^ The Key Laboratory of Biomarker High Throughput Screening and Target Translation of Breast and Gastrointestinal Tumor, Dalian, China; ^3^ Department of Intervention, The Second Hospital Affiliated to Dalian Medical University, Dalian, China

**Keywords:** *BRAF^V600E^* mutation, BM of NSCLC, *BRAF^S365L^* mutation, CSF ctDNA, vemurafenib

## Abstract

*BRAF* mutations, primarily sensitizing mutations, such as *BRAF^V600E^*, have been proven to response to the *BRAF* inhibitor, Dabrafenib combined with trametinib therapy, but there have been no data demonstrating that it has activity against NSCLC-related brain metastases (BM). How patients harboring *BRAF^S365L^* mutation (a rare mutation following *BRAF^V600E^*-inhibitor treatment) in NSCLC is unknown. Vemurafenib, another *BRAF* inhibitor, can reverse the resistance that develops with the *BRAF^S365L^* mutation following dabrafenib combined with trametentinib treatment in melanoma, but none has been reported in NSCLC. Lung papillary cancer, as a rare typing, occupies about 4% of NSCLC. Hence, we reported the first case of a patient with BM of lung papillary carcinoma harboring a *BRAF^V600E^* mutation who benefited from dabrafenib combined with trametinib, and following the development of the *BRAF^S365L^* mutation, vemurafenib remained an effective therapeutic option. Moreover, we found that the next-generation sequencing (NGS) of cerebrospinal fluid (CSF) circulating tumor DNA (ctDNA) may potentially provide more accurate information about intracranial lesions than ctDNA in the blood serum, which will be a better detection method.

## Introduction

In recent years *BRAF^V600E^* (v-Raf murine sarcoma viral oncogene homolog B) mutation occurring in 1% to 2% of patients with NSCLC, has become an important therapeutic target (1, 2). Dabrafenib combined with trametinib therapy has received full approval from the United States Food and Drug Administration (FDA) for the treatment of advanced *BRAF^V600E^*-mutant NSCLC in 2017, but none has reported its activity against NSCLC with BM. Unfortunately, resistance inevitably develops in *BRAF^V600E^*-driven NSCLC patients, such as *BRAF^S365L^* mutation. One study showed that the *BRAF^S365L^* mutation can result in resistance to the MAPK inhibitor trametinib in melanoma with *BRAF^V600E^* mutation, but the resistance can be reversed by vemurafenib (3). Vemurafenib, another selective oral inhibitor of *BRAF^V600E^* kinase, is associated with a response rate of approximately 50% and improved survival among patients with *BRAF^V600E^* mutation-positive metastatic melanoma. However, there is no such research in NSCLC, and a phase II trial of vemurafenib in *BRAF^V600E^*-mutant NSCLC is currently ongoing (4). Lung papillary cancer, as a rare typing of lung adenocarcinoma, occupies about 4% of NSCLC, and provides a better prognosis than lung mucinous adenocarcinoma, but had a worse one than other types of lung adenocarcinoma. And *BRAF^V600E^* may occur in 0.08% of lung papillary cancer, which is quite a small amount of it. Thus, we report, for the first time, the use of dabrafenib combined with trametinib for treating BM of lung papillary cancer carrying the *BRAF^V600E^* mutation, and vemurafenib remained an effective therapeutic strategy for overcoming the resistance induced by the *BRAF^S365L^* mutation, which was identified by genetic sequencing of CSF ctDNA in this patient.

## Case Report

**Figure 1:**
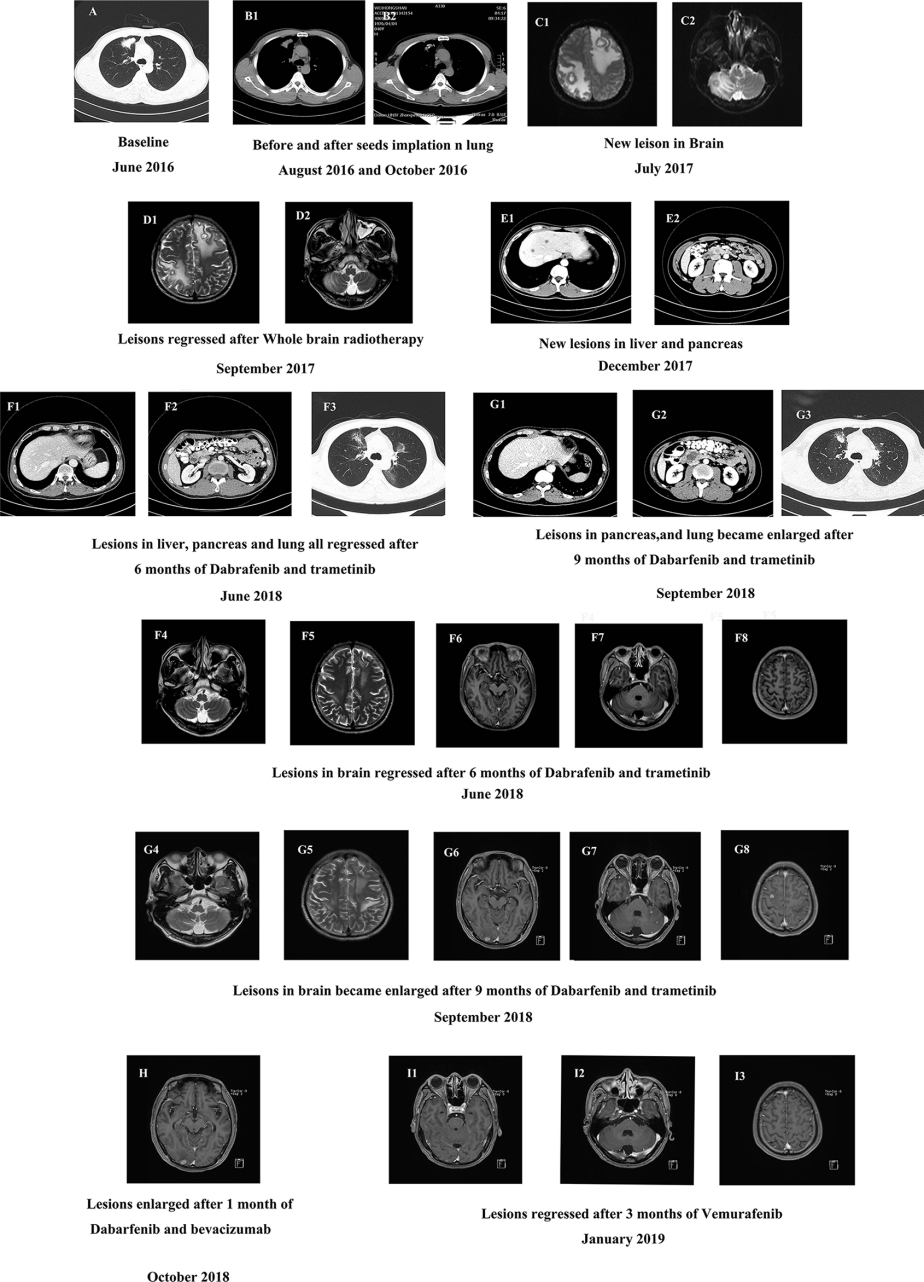
**(A)** The baseline CT scan of the patient’s chest in June 2016. **(B)** CT scan of the chest before and after the seeds implantation. **(C)** The baseline MRI scan of the patient’s brain in July 2017. **(D)** MRI scan of the brain after 2 months of whole brain radiotherapy treatment in September 2017. **(E)** Contrast-enhanced CT scan of the new lesions in abdomen in December 2017. **(F1–8)** CT scan of the abdomen **(F1–2)**, chest **(F3)** and MRI scan of the brain **(F4–8)** after 6 months of dabrafenib combined with trametinib therapy in June 2018. **(G1–8)** CT scan of the patient’s abdomen **(G1–2)**, chest **(G3)** and MRI scan of the brain **(G4–8)** after 9 months of dabrafenib combined with trametinib therapy in September 2018. **(H)** MRI scan of the brain after a month of dabrafenib with bevacizumab therapy in November 2018. **(I)** MRI scan of the brain after 3 months of vemurafenib with bevacizumab therapy in March 2019.

**Figure 2:**
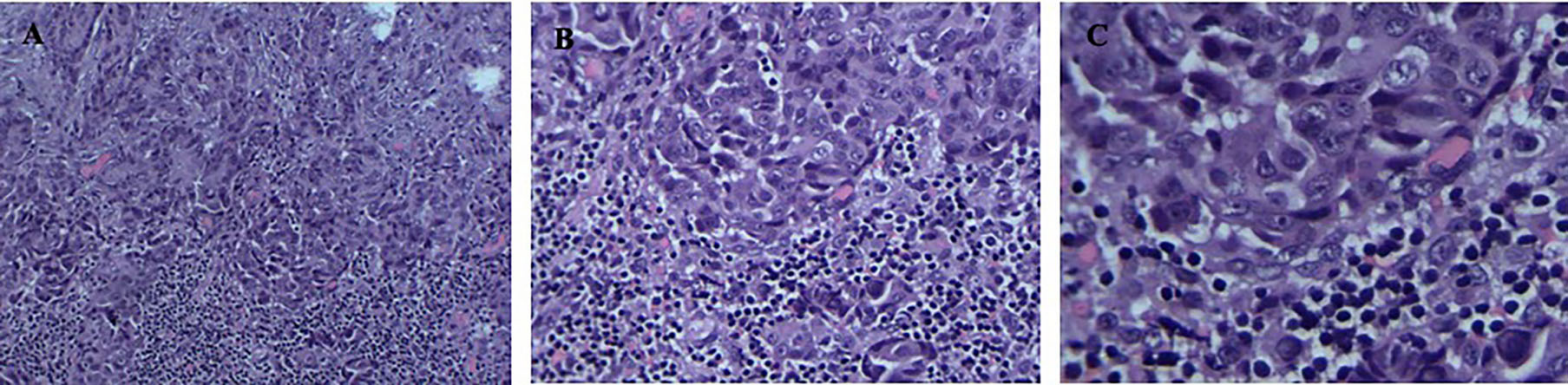
**(A–C)** Positive detection of papillary carcinoma cells in needle biopsy sample of the superficial lymph node biopsy. Magnification; ×100, ×200, ×400.

The patient was a 40-year-old man who presented without any symptoms. But during the routine medical examination, chest X-ray ordered revealed a shadow in his right lung. A subsequent chest computed tomography (CT) scan revealed consolidation in the upper lobe of the right lung (**Figure 1A**). Palpation revealed a mass in the right supraclavicular lymph node. A pathological examination of the superficial lymph node biopsy was conducted, and lung papillary carcinoma was diagnosed (**Figures 2A–C**). The primary lesion was present in the right lung, with right supraclavicular lymph node metastases, and the disease was diagnosed as right lung papillary cancer cT2N3M0(IIIB). The lymph node biopsy revealed the absence of EGFR mutation or ALK fusion by ARMS-PCR, so four cycles of chemotherapy with paclitaxel plus carboplatin were given, and the lesions remained stable, with the curative effect stable disease (SD) and the PFS 3 months. To achieve better results, radioactive seed implantation was performed in the lesion of the right lung, by percutaneous puncture under the guidance of CT scan, 28 seeds were implanted, with the radiation dose 140 Gy, seeds activity 0.6 and the half-life 2.5 months, and the lesions again remained stable, with the curative effect SD and the PFS 9 months (**Figures 1B1**, **2**). Then the patient returned with paroxysmal headache. Head magnetic resonance imaging (MRI) revealed lesions in the right parietal lobe, bilateral frontal lobes, and the right cerebellar hemisphere (**Figures 1C1, 2**). Whole brain radiotherapy was administered, with the whole brain (6MV X ray, Dt:3960cGy/180cGy/22F) and the metastases lesions (6MV X ray, Dt:5280cGy/240cGy/22F). After the radiotherapy, the lesions regressed with the curative effect evaluation was PR (partly response, PR) (**Figures 1D1, 2**). At that time, a chest CT showed that the lesion in the right lung remained stable, but new lesions were observed in the lower lobe of the left lung and the mediastinal lymph nodes, so intensity-modulated radiation therapy was administered to the mediastinal lesions. Three months later, chest CT again showed stable lesions, but an abdominal contrast-enhanced CT revealed multiple lesions in the liver, and a mass in the pancreas metastases (**Figures 1E1, 2**).

**Figure 3:**
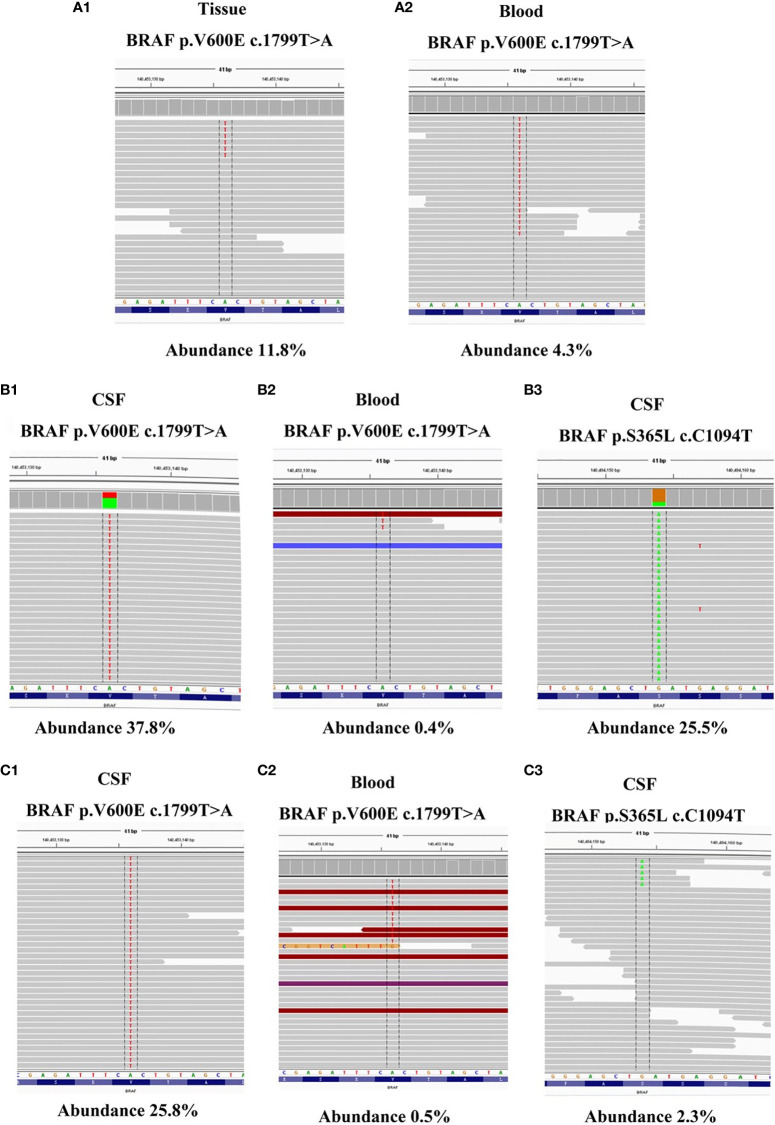
**(A1–2)** NGS reads showing the *BRAF^V600E^* mutation in both tumor tissue *BRAF^V600E^* mutation in both tumor tissue and ctDNA from the plasma were visualized using the Integrated Genomics Viewer before dabrafenib combined with trametinib therapy. **(B1–3)** NGS reads showing the *BRAF^V600E^* and *BRAF^S365L^* mutation in both CSF and ctDNA from the plasma were visualized using the Integrated Genomics Viewer when the resistance of dabrafenib combined with trametinib therapy occurred. **(C1–3)** NGS reads showing the *BRAF^V600E^* and *BRAF^S365L^* mutation in both CSF and ctDNA from the plasma were visualized using the Integrated Genomics Viewer when the resistance of vemurafenib therapy occurred.

To identify further treatment options, targeted next-generation sequencing (NGS) using a cancer-relevant gene panel was performed on both the biopsy sample of the right lung and plasma ctDNA. A *BRAF^V600E^* mutation was identified both in the tissue (allele fraction [AF], 11.8%) and the plasma (AF, 4.3%) ctDNA (**Figures 3A1, 2**). Dabrafenib and trametinib were given orally daily, and the lesions in chest, abdomen and brain all regressed within the first 6 months, with the curative effect evaluation regressed SD (**Figures 1F1–8**), but became enlarged 3 months later (**Figures 1G1–8**), with the curative effect enlarged SD and the PFS 6 months. No evident side effects occurred during the combination therapy. To explore potential resistance mechanisms, targeted NGS of both CSF ctDNA obtained by lumbar puncture and plasma ctDNA was conducted: NGS revealed the presence of the *BRAF^V600E^* mutation in the CSF (AF, 37.8%) and in the plasma (AF, 0.4%) ctDNA (**Figures 3B1, 2**), as well as a newly acquired *BRAF^S365L^* mutation in the CSF (AF, 25.5%) (**Figures 3B3**). The *BRAF^S365L^* mutation indicates resistance to trametinib, so dabrafenib and bevacizumab were given as new therapy, but the lesions in the brain became enlarged, and evaluated as enlarged SD (**Figure 1H**). Therefore, vemurafenib was given with bevacizumab, and the lesions in brain regressed and others remained stable within the first 3 months, with the curative effect evaluation SD (**Figures 1I1–3**). Two months later, the patient experienced convulsions. The lesions in the brain remained stable. Targeted NGS of both the CSF cell-free DNA obtained by lumbar puncture and the plasma ctDNA revealed the presence of the *BRAF^V600E^* mutation in the CSF (AF, 25.8%) and in the plasma (AF, 2.3%) ctDNA (**Figures 3C1, 2**), but the *BRAF^S365L^* mutation in the CSF decreased substantially (AF, 0.5%) (**Figures 3C3**). Therefore, it may be the time of the tolerance of vemurafenib. The PFS of vemurafenib was 5 months, during that time, moderate rash was found in the upper limbs, no drug was given. And the rash disappeared soon after the drug’s withdrawal. Therefore, dabrafenib, trametinib, and bevacizumab were administered again, the symptoms of dizzy, vomiting, and convulsions relieved in the first few months, and the patient died 8 months later.

## Discussion

*BRAF* mutation is important event in cancer development. Mutations in the BRAF gene are referred to as “activating mutations.” *BRAF* proteins are part of a signaling pathway (RAF-MEK-ERK) that affects cell growth in several ways, to promote cell proliferation, cell survival, and aid in differentiation, migration, and inhibits apoptosis in several cancers, which has been proved to be a higher risk of recurrence and a bad prognostic factor of cancer. *BRAF^V600E^* mutation, as the main type of *BRAF* mutations, has been proven to participate in several cancers, such as melanoma, NSCLC, colorectal cancer, and thyroid cancer. There have been three main *BRAF* inhibitors at present, dabrafenib, vemurafeinib, and encorafenib. Dabrafenib is a small molecule inhibitor of the *BRAF*-mutant kinase family, as a potent ATP-competitive inhibitor of *BRAF* kinase, it was approved for use in monotherapy or in combination with trametinib for the treatment of unresectable or metastatic *BRAF^V600E^* -mutated melanoma, advanced *BRAF^V600E^* -mutated NSCLC, and *BRAF^V600E^* -mutated locally advanced or metastatic ATC (anaplastic thyroid cancer, ATC). Vemurafenib, as another inhibitor of the *BRAF* mutant family, is known to promote the apoptosis of mutated cells in a dose-dependent manner. Specifically, it interrupts the BRAF/MEK step in the BRAF/MEK/ERK pathway. It was approved by the FDA in patients with *BRAF^V600E^* -mutated melanoma or the Erdheim-Chester disease containing the *BRAF^V600E^* mutation. In melanoma, BRAF monotherapy (vemurafenib) occurs drug resistance in a short time, and the combination therapy of dabrafenib and trametinib simultaneously inhibits the upstream and downstream pathway of MAPK, increases the efficacy and safety of monotherapy with *BRAF* inhibitor, and significantly improves the prognosis of patients with *BRAF* mutant melanoma.

In NSCLC, dabrafenib combined with trametinib therapy has received full FDA approval for the treatment of advanced *BRAF^V600E^*-mutant NSCLC. The mPFS, OS, DCR, and ORR in first line of therapy by dabrafenib combined with trametinib may get 10.8 months, 17.3 months, 75%, and 63.9%. And the data will be 10.2 months, 18.2 months, 80.7% and 68.4% in subsequent lines of therapy. Compared with the Dabfrafenib monotherapy, the combination can inhibit the BRAF/MEK/ERK pathway completely. ORR rate for the combination had increased to 67% as did the median progression free survival, 10.2 months. Median overall survival was reported as 12.7 months in the Dabrafenib only cohort versus 18.2 months in the Dabrafenib and Trametinib cohort. Vemurafenib is another significant *BRAF* inhibitor, several clinical trials have indicated that it can be used in NSCLC harboring *BRAF^V600E^* mutation with the ORR 42% and median progression free survival was 7.3 months, which has been proved to be inferior to the combination. And it may be the reason for the failure to be approved by FDA in the NSCLC therapy. But there are no data that demonstrate that it has activity against NSCLC-related BM (5). The related clinical studies explored the patients with NSCLC harboring *BRAF^V600E^*, to show that the combination therapy of dabrafenib and trametinib would be the first line therapy, but the studies didn’t analyses the cases with brain metastases or not. However, in melanoma-related brain metastases, some clinical trials, such as the (COMBI-MB): a multi-cohort, open-label, phase 2 trial has showed dabrafenib and trametinib was active with a manageable safety profile in patients with *BRAF^V600E^* –mutation, but the median duration of response was relatively short (6). These results provide evidence of clinical benefit with dabrafenib and trametinib and support the need for additional research to further improve outcomes in patients with melanoma with brain metastases. Based on its effectiveness in the treatment of melanoma with BM, combination dabrafenib/trametinib therapy was given for this patient with lung papillary cancer with BM, and the lesions remained regressed for 6 months.

However, resistance developed, and the lesions became enlarged. Targeted NGS revealed the presence of the *BRAF^V600E^* mutation in the CSF (AF, 37.8%) and in the plasma (AF, 0.4%) ctDNA, and a newly acquired *BRAF^S365L^* mutation was revealed in the CSF (AF, 25.5%). The N-terminal, S365, is removed in BRAF V600E splice variants but its importance in full-length *BRAF^V600E^* mutants remains uncertain. *BRAF^V600E^* S365L displayed reduced sensitivity to RAF inhibitor at the level of MEK-ERK1/2 signaling, cell growth, and cell viability, suggesting that alteration or removal of the S365 14-3-3 binding site may contribute to RAF inhibitor resistance (7). To the best of our knowledge, there have been no studies of *BRAF^S365L^* mutation reported in NSCLC with BM. But one study suggested that *BRAF^S365L^* mutation indicates resistance to the MAPK inhibitor trametinib in melanoma with *BRAF^V600E^* mutation, but this resistance can be reversed by vemurafenib (3). Vemurafenib, another selective oral inhibitor of *BRAF^V600E^* kinase, is associated with a response rate of approximately 50% and improved survival among patients with *BRAF^V600E^* mutation-positive metastatic melanoma (8). Additionally, in a single-arm phase II study of vemurafenib for metastatic melanoma patients with BM, survival was extended to over 6 months, which is longer than the expected natural course of patients with BM (< 3 months) (9). However, there are no such reports in NSCLC, and a phase II trial of vemurafenib in *BRAF^V600E^*-mutant NSCLC is currently ongoing (4). In theory, the CNS efficacy of these drugs is based on the rationale that the blood-brain barrier is disrupted by tumor activity. In this case, with the patient’s approval, vemurafenib was given, and the lesions regressed within the first 3 months and remained stable for the following 2 months. Then, the disappearance of the *BRAF^S365L^* mutation was noted, and dabrafenib and trametinib therapy was given, the symptoms of dizzy, vomiting and convulsions relieved in the first few months, to prove it benefit from the therapy.

It is worth mentioned that we confirmed that gene alterations detected in CSF ctDNA were superior to those of plasma ctDNA in *BRAF*-mutant NSCLC with BM. Owing to the blood-brain barrier, it is difficult for CSF ctDNA to circulate fully within the blood system, which results in a limited amount of ctDNA from the CNS being released to plasma. Therefore, plasma cannot accurately represent intracranial lesions clearly. From several papers of NSCLC-related brain metastases, the detection rate of CSF ctDNA varying from about 57.1% to 80.0%, and in blood ctDNA detection the rate was from 21.3% to 23.8% (10–12).When the resistance developed, targeted NGS revealed the presence of the *BRAF^V600E^* mutation in the CSF (AF, 37.8%) and in the plasmactDNA (AF, 0.4%). But the newly acquired *BRAF^S365L^* mutation was only revealed in the CSF (AF, 25.5%), suggesting the importance of CSF ctDNA as a liquid biopsy source for BM (10).

In addition, bevacizumab was used in this patient. As we know, anti-angiogenic therapy plays a major role in the management of brain metastases in NSCLC. Bevacizumab is widely used in combination with chemotherapy, radiotherapy, EGFR-TKI targeted therapy, and immunotherapy for NSCLC with BM. In a retrospective cohort study of bevacizumab for advanced NSCLC, analysis of the brain metastases subgroup showed that bevacizumab plus carboplatin paclitaxel significantly prolonged OS compared with carboplatin paclitaxel alone (11.3 months vs. 2.3 months). In this case, the benefit from bevacizumab seemed to be indistinct, several studies of bevacizumab combination therapy for NSCLC with BM are still in progress.

In summary, this is the first case report to describe a patient with *BRAF^V600E^*-mutated lung papillary cancer with BM responding to dabrafenib combined with trametinib therapy; additionally, vemurafenib was effective against the newly evident *BRAF^S365L^* mutation. This case gives support for ongoing trials investigating the role of *BRAF* inhibitors in *BRAF*-mutated NSCLC with BM. And the findings also suggest that genome sequencing of the CSF is much more accurate than that of ctDNA in blood serum in NSCLC with BM. If ctDNA detection in blood serum yields negative results, CSF may be used to enhance detection.

## Data Availability Statement

The original contributions presented in the study are included in the article/supplementary material. Further inquiries can be directed to the corresponding authors.

## Ethics Statement

The study involving human participants was reviewed and approved by the Affiliated Zhongshan Hospital of Dalian University Biomedical Research Ethics Committee. The patients/participants provided their written informed consent to participate in this study. Written informed consent was obtained from the individual(s) for the publication of any potentially identifiable images or data included in this article.

## Author Contributions

JG and GW performed the radiological analysis of MRI CT and PET-CT images. JJ wrote the first draft of the manuscript. RW wrote sections of the manuscript. All authors contributed to the article and approved the submitted version.

## Funding

This work was supported by grants from the National Science Foundation of China (#81803109) and the Doctor Study-up Foundation of Liaoning Province (2019-BS-010).

## Conflict of Interest

The authors declare that the research was conducted in the absence of any commercial or financial relationships that could be construed as a potential conflict of interest.

## References

[1] Planchard D Smit EF Groen HJM Mazieres J Besse B Helland A. Dabrafenib Plus Trametinib in Patients With Previously Treated *BRAF V600E* Mutant Metastatic Non-Small Cell Lung Cancer: An Open-Label, Multicentre Phase 2 Trial. Lancet Oncol (2016) 17:984–93. 10.1016/S1470-2045(16)30146-2 PMC4993103 27283860

[2] Paik PK Arcila ME Fara M Sima CS Miller VA Kris MG. Clinical Characteristics of Patients With Lung Adenocarcinomas Harboring BRAF Mutations. J Clin Oncol (2011) 29:2046–51. 10.1200/JCO.2010.33.1280 PMC3107760 21483012

[3] Hyman DM Puzanov I Subbiah V Faris JE Chau I Blay J-Y. Vemurafenib in Multiple Nonmelanoma Cancers With *BRAF* V600 Mutations. N Engl J Med (2015) 373(8):726–36. 10.1056/NEJMoa1502309 PMC4971773 26287849

[4] Subbiah V Gervais R Riely G Hollebecque A Blay J-Y Felip E. Efficacy of Vemurafenib in Patients With Non–Small-Cell Lung Cancer With BRAF V600 Mutation: An Open-Label, Single-Arm Cohort of the Histology-Independent Ve-Baskets. Am Soc Clin Oncol (2020) 3:1–9. 10.1200/PO.18.00266. PMC7446432 32914022

[5] Planchard D Maziers J Riely GJ. Interim Results of Phase II Study BRF 113928 of Darafenib in BRAF V600E Mutation-Positive non-Small Cell Lung Cancer(NSCLC) Patients. J Clin Oncol (2013) 31:15–24. 10.1200/jco.2013.31.15_suppl.8009

[6] Davies MA Saiag P Robert C Grob J-J Flaherty KT Arance A. Dabrafenib Plus Trametinib in Patients With BRAF-mutant Melanoma Brain Metastases (COMBI-MB): A Multicentre, Multicohort, Open-Label, Phase 2 Trial. Lancet Oncol (2017) 18:863–73. 10.1016/S1470-2045(17)30429-1 PMC5991615 28592387

[7] Vido Michael J Justin R Aplin Andrew E. Role of Serine 365 in BRAF V600E Sensitivity to RAF Inhibition. Pigment Cell Melanoma Res (2020) 1–7. 10.1111/pcmr.12932 PMC8285005 33000894

[8] Robinson SD Joyce A O’Shaughnessy C Cowey L Konduri K. Braf V600E-Mutated Lung Adenocarcinoma With Metastases to the Brain Responding to Treatment With Vemurafenib. Lung Cancer (2014) 85(2):326–30. 10.1016/j.lungcan.2014.05.009 24888229

[9] Mcarthur G Maio M Arance A Nathan P Blank C Avril MF. Vemurafenib in Metastatic Melanoma Patients With Brain Metastases: an Open-Label, Single-arm, Phase 2, Multicenter Study. Ann Oncol (2017) 28(3):634–641. 10.1093/annonc/mdw641 27993793

[10] De Mattos-Arruda L Mayor R Ng CKY Weigelt B Torrejon D Oliveria M. Cerebrospinal Fluid-Derived Circulating Tumour DNA Better Represents the Genomic Alterations of Brain Tumours Than Plasma. Nat Commun (2015) 6:8839–45. 10.1038/ncomms9839 PMC5426516 26554728

[11] Ge M Zhan Q Ji X Zhou X Huang R Liang X. Different Next-Generation Sequencing Pipelines Based Detection of Tumor DNA in Cerebrospinal Fluid of Lung Adenocarcinoma Cancer Patients With Leptomeningeal Metastases. BMC Cancer (2019) 19:143. 10.1186/s12885-019-5348-3 30755180 PMC6373107

[12] Chunhua M Xueling Y Wenge X Yu H. Detection of Circulating Tumor DNA From Non-Small Cell Lung Cancer Brain Metastasis in Cerebrospinal Fluid Samples. Thorac Cancer (2020) 11:588–93. 10.1111/1759-7714.13300 PMC7049513 31944608

